# Modifications of Antiepileptic Drugs for Improved Tolerability and Efficacy

**Published:** 2008-02-14

**Authors:** Cecilie Johannessen Landmark, Svein I. Johannessen

**Affiliations:** 1 Cecilie Johannessen Landmark, Associate Professor, Dept. of Pharmacy, Faculty of Health Sciences, Oslo University College, Pilestredet 50, N-0167 Oslo, Norway; 2 Svein I. Johannessen, Director of Research. The National Center for Epilepsy, Sandvika, Division of Clinical Neuroscience, Rikshospitalet University Hospital, Oslo, Norway

**Keywords:** antiepileptic drugs, chemical modification, efficacy, monitoring, pharmacokinetics, pharmacodynamics, tolerability

## Abstract

**Introduction:**

A large number of antiepileptic drugs (AEDs) are available today, but they may not be satisfactory regarding clinical efficacy, tolerance, toxicity or pharmacokinetic properties. The purpose of this review is to focus upon the rationale behind the chemical modifications of several recently marketed AEDs or drugs in development and to categorize them according to the main purposes for the improvements: better efficacy or tolerability accompanied by improved pharmacokinetic properties.

**Material and Method:**

AEDs that have been chemically modified to new derivatives during the last years are reviewed based on recent publications and PubMed-searches.

**Results and Discussion:**

Improvement in pharmacokinetic parameters may affect both tolerability and efficacy. Modifications to improve tolerability include various valproate analogues, divided into aliphatic amides, cyclic derivatives or amino acid conjugates. Furthermore, there are the carbamazepine analogues oxcarbazepine and eslicarbazepine, the felbamate analogues fluorofelbamate and carisbamate (RWJ 33369), and the lamotrigine analogue JZP-4. The levetiracetam analogues brivaracetam and seletracetam and the derivatives of gabapentin, pregabalin and XP13512, have improved selectivity compared to their parent compounds. Other new drugs have new mechanisms of action related to GABA and glutamate receptors; the glutamate antagonists like topiramate (talampanel and NS-1209), and GABA_A_ receptor agonists, benzodiazepine or progesterone analogues (ELB-139 and ganaxolone).

**Conclusion:**

Further challenges for development of new AEDs include investigations of target molecules affected by pathophysiological processes and detailed structure-activity relationships with focus on stereoselectivity. These potential drugs may become of importance in future drug therapy in epilepsy and other CNS disorders.

## Introduction

A variety of antiepileptic drugs (AEDs) are available today, but still there is a need for new drugs, since the existing ones are not satisfactory regarding clinical efficacy, tolerance, toxicity or pharmacokinetic properties. A new generation of the existing AEDs is in development, undergoing preclinical and clinical trials. These compounds have to be more potent, safer, and possess favorable pharmacokinetics to become a successful second-generation of AEDs (Bialer, 2006).

It is important to emphasize that AEDs consist of a heterogenous group of drugs with various chemical properties, such as lipophilicity, ionization, and chirality. They have different chemical structures, and they may affect different target proteins (receptors, enzymes, transporters or voltage-gated ion channels). The seizure types are different in their origin, and patients suffering from epilepsy consist of a heterogenous group, phenotypically and genotypically different.

Both established and new AEDs are in clinical use or undergoing clinical trials in several psychiatric and neurological disorders other than epilepsy. These include migraine, neuropathic pain, bipolar disorder, mania, schizophrenia, anxiety, tremor, and still other conditions are under investigation (for review, see Rogawski and Löscher, 2004a; Spina and Perugi, 2004; Stefan and Feuerstein, 2007; Johannessen Landmark, 2007a). The action of AEDs at the molecular level is incompletely understood, and most AEDs probably have more than one mechanism of action, each of which may contribute to the therapeutic efficacy to a variable extent depending on the actual condition (Perucca, 2004; Johannessen Landmark, 2007a). It is therefore difficult to divide the drugs into specific categories regarding their mechanisms of action and clinical efficacy.

The purpose of the present review is to focus upon the rationale behind the chemical modifications of several recently marketed AEDs or drugs in development and to categorize them according to the main purposes for the improvements; namely better efficacy or tolerability. These modifications are often also accompanied by improved pharmacokinetic properties.

## Material and Methods

This review is based upon recent publications and PubMed searches on AEDs. The marketed AEDs included are carbamazepine, felbamate and fluorofelbamate, gabapentin, lamotrigine, levetiracetam, oxcarbazepine, pregabalin, topiramate, and valproate. AEDs in development included brivaracetam, eslicarbazepine (BIA 2-093), ELB139, JZP-4, NS1209, carisbamate (RWJ 33369), talampanel, XP13512, and valproic acid derivatives (propylisopropyl acetamide (PID), 2,2,3,3- tetramethylcyclopropanecarbonylurea (TMCU), N-methyl-2,2,3,3-tetramethylcyclopropylamide (MTMCD), valnoctamide and valrocemide). The older AED phenytoin with its derivative fosphenytoin was not included, since this derivative has existed for almost ten years and the clinical use is limited to injections used in the treatment of status epilepticus.

Search terms used were the AEDs mentioned above, anticonvulsant, epilepsy, mechanism of action, efficacy, tolerability, and pharmacokinetic properties, and combinations of these terms.

Relevant peer-reviewed articles in recognized, creditable international journals in English, from the earliest relevant data, 1983–2007, were included in the review. Primary sources were preferred, but review articles of specific importance were also included. Abstracts, unpublished or non-English material, and papers of limited relevance or out-of-date results or choice of methods were excluded.

## Strategies for Modifications and Monitoring of AEDs

**Figure 1:**
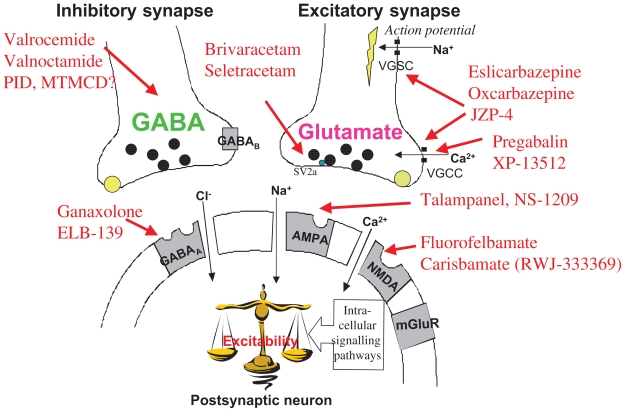
Main proposed mechanisms of action of newer antiepileptic drugs (AEDs) in the inhibitory GABAergic and the excitatory glutamatergic synapse. The black spots are reuptake proteins for GABA and glutamate (two distinct, selective proteins). The grey receptor sites are metabotropic receptors, GABA_B_ for GABA and mGluR, for glutamate. **Abbreviations:** SV2A: synaptic vesicle protein 2A, the specific binding site for levetiracetam; VGCC and VGSC are voltage-gated calcium- and sodium channels, respectively. (Modified from Johannessen Landmark, 2007).

**Figure 2:**
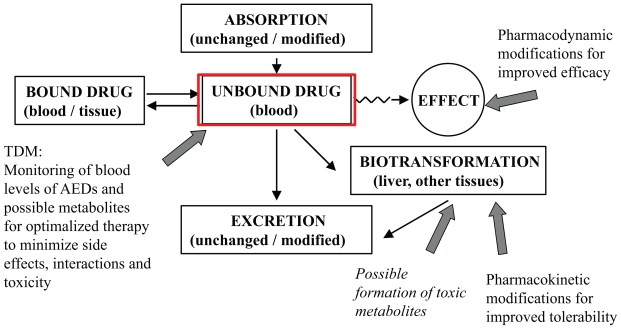
Pharmacokinetic paramaters. A drug is absorbed to the systemic circulation, where the drug may be bound to plasma proteins or distributed throughout body tissues (fat or fluid). It is only the free fraction of the drug in plasma that can exert an effect. From the systemic circulation the drug is transported to its site of action to exert its pharmacodynamic effect. The drug is undergoing elimination via biotransformation through metabolic pathways and elimination (often through liver, kidneys and faeces). The block arrows point to modifications in efficacy by pharmacodynamic (mechanism of action) factors, and tolerability by modifications of pharmacokinetic parameters.

Most AEDs have several mechanisms of action, and focus has been on designing new AEDs with specific mechanisms of action, or more selective effects on known target molecules for pharmacodynamic effects (Fig. 1). The major improvements of the new derivatives are listed in Table 1. Chemical modifications resulting in a better pharmacokinetic profile have been emphasized for improving AEDs, resulting in better tolerability and efficacy (Fig. 2).

Basic pharmacokinetic parameters are the factors in the body that affects the drug from the time it is taken, including absorption, distribution, metabolism and excretion (Fig. 2). A drug is absorbed to the systemic circulation, where it may be bound to plasma proteins or distributed throughout body tissues (fat or fluid). From the systemic circulation the free fraction (which may be neutral or ionized) of the drug is transported to its site of action to exert its pharmacodynamic effect. The drug is undergoing elimination via biotransformation through metabolic pathways and elimination (often through liver, kidneys and faeces). Other parameters derived from these basic processes are e.g. bioavailability and half-life, which affect the efficacy of the drug, to which extent it reaches the site of action and how long it remains in the systemic circulation. A prolonged half-life of a drug or changed distribution in various body tissues may affect the efficacy of the drug. Therapeutic drug monitoring (TDM) is a useful tool also in the treatment of patients using older as well as newer AEDs (Johannessen and Tomson, 2002, 2006) Monitoring of AEDs gives the opportunity to observe pharmacokinetic and pharmacodynamic interactions, to avoid side effects and toxic levels and active metabolites in the blood (Fig. 2).

In the following, each of the parent drugs will be briefly described, regarding pharmacological characteristics, including mechanism of action and clinical use and limitations. Chemical characteristics and modifications will then be described and discussed.

## Improved Tolerability of New AEDs

The tolerability of AEDs has often been improved by modifications of pharmacokinetic properties to reduce potential toxic metabolic pathways or teratogenic effects, as for valproate, carbamazepine, and felbamate (Fig. 1).

### Modifications of valproic acid

#### Parent drug: Valproic acid/valproate

##### Proposed mechanisms of action

Valproic acid or valproate, its corresponding base which most often is used in drug formulation, is a broad spectrum AED. It has been investigated through the past four decades with demonstrated effects in a variety of elecrophysiological, neurochemical, seizure and animal models, and several mechanisms of action have been proposed. Attention has been drawn to effects on enhancement of GABAergic neurotransmission, where valproate affects several enzymes related to GABA synthesis and degradation (Löscher, 2002a, b; Johannessen and Johannessen, 2003; Johannessen Landmark, 2007b). The effect of valproate on voltage-gated sodium channels, in addition to potassium and calcium channels, is no longer regarded as an important clinically relevant mechanism of action (Löscher, 2002a, b; Johannessen et al. 2001; Johannessen and Johannessen, 2003).

In addition, valproate is involved in modulation of intracellular signalling pathways. Effects on these intracellular proteins are regarded as important for the understanding of the pathophysiology of bipolar disorder as well as the efficacy of valproate in bipolar disorder (Rogawski and Löscher, 2004a, b; Anmann and Grünze, 2005; Johannessen Landmark, 2007a, b). Intracellular targets include modulation of inositol metabolism, kinases as ERK and MARCK, glycogen synthase-3, protein kinase C, and early inducible genes (Göttlicher et al. 2001; Phiel et al. 2001; Löscher, 2002a, b; Johannessen and Johannessen, 2003; Harwood and Agam, 2003; Ju and Greenberg, 2003; Brunello and Tascedda, 2003; Anmann and Grünze, 2005). Altered expression of a number of different genes expressed following valproate treatment in mice, indicates that valproate regulates a large number of different functional pathways in the brain (Chetcuti et al. 2005).

##### Clinical use

Valproate is widely used in a number of neurological and psychiatric disorders today. It is extensively used in epilepsy and is effective in all seizure types, in addition to neuropathic pain, migraine, and bipolar disorder (Rogawski and Löshcer, 2004a; Spina and Perugi, 2004; Johannessen Landmark, 2007a; Anmann et al. 2007). Valproate is under investigations for further extensive clinical use, as in schizophrenia, neuroprotection and cancer (Dou et al. 2003; Eyal et al. 2005; Ichikawa et al. 2005; Yeow et al. 2006). Since valproate has several proposed mechanisms of action, it is regarded as a broad-spectrum drug with a wide therapeutic potential.

A fatale adverse effect of valproate is hepatotoxicity, its toxic metabolite being 4-ene valproate (Bailie, 1992). Children less than 2 years old receiving Valproate in polytherapy and suffering from metabolic genetic disorders appear to be at highest risk for developing fatal hepatic dysfunction (1/600) (Bryant and Dreifuss, 1996). Another restriction in the clinical use of valproate is the risk of teratogenicity. The incidence of congenital malformations in the offspring of mothers treated with valproate during pregnancy is 3 to 4 times higher than in offspring not exposed to valproate (Kaneko et al. 1999; Tomson et al. 2004; Perucca, 2005; Aldenkamp et al. 2006).

#### Valproic acid analogues

A number of analogues have been investigated, and several of them have been designed as stable amide derivatives of valproic acid with no acidic function or minimal conversion to the acid (Bialer and Yagen, 2007). Three strategies have been implemented in this attempt, the development of aliphatic amide analogues, cyclic derivatives, and amino acid conjugates, (Bialer, 2006; Bialer and Yagen, 2007).

**Figure 3:**
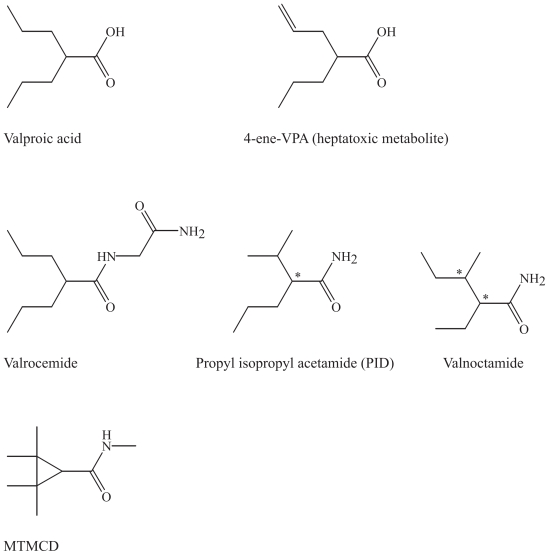
Valproic acid, its toxic metabolite and several of the valproic acid analogues.

Some of the main derivatives will be briefly presented including valrocemide (valproyl glycinamide), the tetramethylcyclopropyl analogue (TMCU) and isovaleramide (NPS 1776) (Isoherränen, 2001a, b; Bialer et al. 2006; Sobol et al. 2006) (Fig. 3). The valproic acid amide derivatives, valnoctamide, diisopropylacetamide, and N-methyl-2,2,3,3- tetramethylcyclopropylamide (MTMCD) show more potent antiallodynic effects than valproic acid and exert minimal motor and sedative side effects at analgesic doses in a rat model of neuropathic pain and may consequently, become new drugs for the treatment of neuropathic pain (Winkler et al. 2005a, b). Investigations of the clinically relevant mechanisms of these second-generation substances are still ongoing. Valrocemide is undergoing phase II in clinical development (Bialer et al. 2006).

### Chemical characteristics and modifications of valproic acid and its derivatives

Valproic acid (di-n-propyl acetic acid) or valproate, its corresponding base, and its simple structure of a short, branched fatty acid differs to a great extent from the substituted heterocyclic ring structures characterizing other older AEDs. Some of its derivatives are illustrated for comparison with the simple structure of the parent drug (Fig. 3).

#### Aliphatic amide analogues and amino acid conjugates

The amide derivatives are more potent than valproic acid and their corresponding acids, and a series of structure-pharmacokinetic-pharmacodynamic relationships have been studied (Winkler, 2005a, b; Sobol et al. 2006; Bialer, 2006).

In the simple structure of PID, the molecule is an acetamide, but the propyl isopropyl skeleton is present, resulting in one chiral center. It is the corresponding amide of valproic acid. In valrocemide, a glycinamide is attached to the carboxylic acid end of the molecule. In valnoctamide the carboxylic acid in vaplroic acid is replaced here by an amide like in PID, and one methyl group is reorganized to achieve two chiral centra. Racemic valnoctamide and the stereoisomers (2R,3S)- and (2S,3S) valnoctamide (with the best brain penetration) were demonstrated to be effective anticonvulsants in animal models of partial seizures and were more potent than valproic acid (Isoherrränen et al. 2003). It seems reasonable that only one pair of the racemate is pharmacologically active, since biological complexing usually is stereoselective.

#### Cyclic derivatives

N-methyl-2,2,3,3- tetramethylcyclopropylamide MTMCD (MTMCD) is a cyclopropylamide with four methyl groups, and several other cyclic derivatives have been developed, as TMCU (Bialer, 2006; Sobol et al. 2006). TMCU is similar to MTMCD but has an amide attached. These cyclic analogues possess two quanternary carbons in the β-position and cannot be biotransformed into the minor metabolites of valproic acid, 4-ene-VPA and 2,4-diene-VPA with a terminal double bond, which is presumed to be the source of hepatotoxicity (Konig et al. 1999; White et al. 2002; Sobol et al. 2005).

### Modifications of carbamazepine

#### Parent drug: Carbamazepine

##### Proposed mechanism of action

The main action of carbamazepine is mediated through inhibition of voltage-activated sodium channels and consequently, inhibition of action potentials and excitatory neurotransmission. High frequency, repetitive neuronal firing is therefore limited (Kuo, 1998; Kuo et al. 2004). The inhibitory potency is strongly use-dependent and accumulates with prolonged activation (Rogawski and Löscher, 2004b). This is a classic mechanism of action for AEDs that is also shared by e.g. the older drug phenytoin, and several other AEDs as one of more mechanisms of action. In addition, as for valproate modulation of intra-cellular signalling pathways has been shown to be important for understanding of the pathophysiological background for bipolar disorder (Rogawski and Löscher, 2004a; Anmann and Grünze, 2005).

#### Oxcarbazepine

Oxcarbazepine differs from carbamazepine as the drug inhibits several types of voltage-gated calcium channels (Ahmad et al. 2005). Also for oxcarbazepine, modulation of intracellular signalling pathways is important for the pathophysiological background for bipolar disorder (Rogawski and Löscher, 2004a; Anmann and Grünze, 2005). Oxcarbazepine is a non-toxic derivative of carbamazepine with a reduced drug interaction potential (Bialer et al. 2004; Schmidt and Elger, 2004).

##### Clinical use of carbamazepine and oxcarbazepine

Oxcarbazepine has been available in Europe for many years but only recently in the US. Carbamazepine and oxcarbazepine are widely used in several neurological and psychiatric disorders. Carbamazepine is regarded as a first-line drug in epilepsy, and it is also frequently used in neuropathic pain and bipolar disorder (Rogawski and Löscher, 2004a; Spina and Perugi, 2004; Johannessen Landmark, 2007a; Bialer et al. 2007; Anmann et al. 2007). Both drugs are broad-spectrum drugs, based on their mechanisms of action and wide clinical use.

The goal for developing the derivatives of carbamazepine has been to avoid the potentially toxic epoxide-metabolite skin rash and less susceptibility to pharmacokinetic interactions, as it does not undergo inducible cytochrome CYP3A4-mediated oxidative metabolism in the liver (Bialer, 2006).

#### Eslicarbazepine (BIA 2-093)

The carbamazepine analogue eslicarbazepine acetate, BIA 2-093, (S9-(−)-10-acetoxy-10,11-dihydro-5H-dibenz/b,f/azepine-5-carboxamide was designed for improved efficacy and safety (Bialer et al. 2007). BIA 2-093 is a derivative of carbamazepine and oxcarbamazepine, and is a prodrug for the main active metabolite (S)-licarbazepine (Bialer et al. 2007). The compound is currently undergoing clinical phase III trials in epilepsy and phase II trials in bipolar disorder (Bialer et al. 2007).

#### Chemical characteristics and modifications of carbamazepine and its derivatives

**Figure 4:**
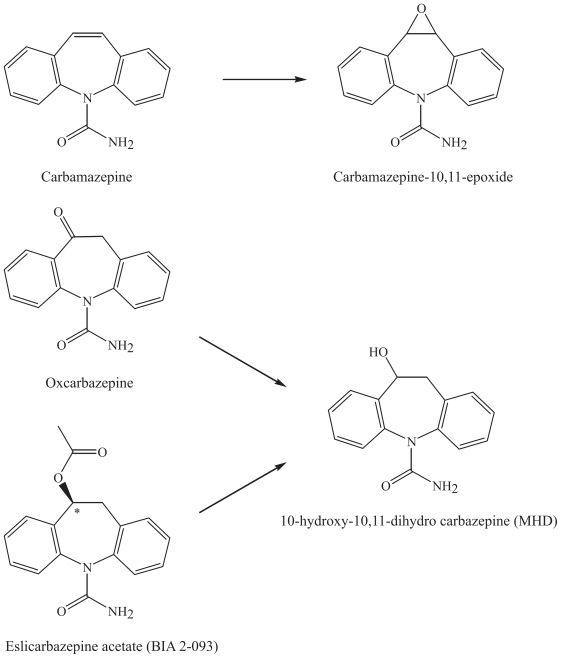
Metabolism of carbamazepine and its derivative oxcarbazepine and eslicarbazepine.

Carbamazepine is a dibenzoazepine, a carbamate, its main metabolite being the 10,11-epoxide. This unstable epoxide is not toxic, in contrast to other epoxides that are formed after enzymatic degradation in the liver (Fig. 4). In the structure of its derivative oxcarbazepine, there is addition of a ketone group on the N-containing cyclic structure. Oxcarbazepine is a prodrug for the main active metabolite, mono-hydroxy derivative (MHD), 10-hydroxy-10,11-dihydro carbazepine, that can be measured in the blood for therapeutic drug monitoring purposes (Johannessen and Tomson, 2006). BIA 2-093 is a dibenzazepine derivative of carbamazepine and oxcarbamazepine, and is a prodrug for the main active metabolite (S)-licarbazepine, which is one of the enantiomers of MHD (Fig. 4).

### Modifications of felbamate

#### Parent drug: Felbamate

##### Proposed mechanisms of action

Felbamate is regarded as a broad-spectrum AED with several proposed mechanisms of action, including antagonism at the glutamatergic NMDA receptor at the NR2 subunit, in clinically relevant concentrations (Kuo et al. 2004). Other mechanisms of action include inhibition of voltage-gated sodium and calcium channels (Rogawski and Löscher, 2004b).

##### Clinical use

After serious adverse events including aplastic anemia and liver failure caused by production of a reactive metabolite of felbamate in a limited number of the patient population, the use of the drug was thoroughly considered. The clinical use of felbamate is now limited and is only used in some forms of refractory epilepsy and Lennox Gastaut syndrome in children (Perucca, 2004).

#### Fluorofelbamate

A non-toxic analogue, fluorofelbamate, 2-phenyl-2-fluoro-1,3-propanediol dicarbamate, was developed due to the serious adverse effects of felbamate. Its mechanisms of action cannot be completely explained by either interactions at glutamate receptor sites or sodium channels (Wallis et al. 2000). It protects against ischemia and hypoxia *in vitro* and *in vivo* (Wallis et al. 2000). Fluorofelbamate is undergoing preclinical trials, and has shown to be effective in a rat model of status epilepticus (Mazarati et al. 2002; Bialer et al. 2007). Pre-clinical findings suggest that fluorofelbamate is not metabolized to the known reactive metabolite of felbamate, where possibly the upper amide group is cleaved off the molecule to give felbamate 2-phenylpropanal (ATPAL) (Parker et al. 2005). The fluor atom will protect the amide groups by its inductive effect, as the size of the ion radius of fluor compared to hydrogen is similar.

#### Carisbamate (RWJ-333369)

Another derivative of felbamate is carisbamate, (S)-2-O-carbamoyl-1-o-chlorophenyl-ethanol. Carisbamate (RWJ-333369) is undergoing phase II and III clinical trials and seems to be well tolerated (Bialer, 2006; Bialer et al. 2007). The compound has been tested in several preclinical models and has a favorable profile in epilepsy models, such as corneal kindling, hippocampus kindling, a genetic absence epilepsy rat model (GAERS) and chemically induced seizures (Bialer et al. 2007).

#### Chemical characteristics and modifications of felbamate and its derivatives

**Figure 5:**
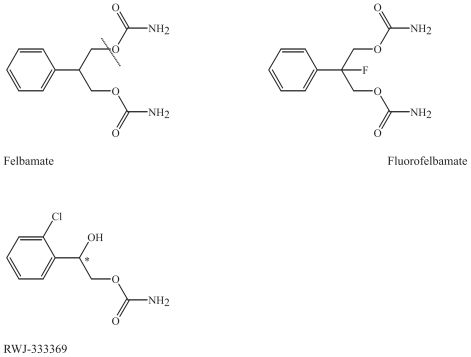
Felbamate and its derivatives, fluorofelbamate and carisbamate (RWJ 33369). The cleavage of felbamate to the toxic metabolite ATPAL is illustrated by the dashed line.

Felbamate is a symmetrical molecule with a benzene ring structure attached to a central carbon atom with a diether binding to two amide groups (Fig. 5). In fluorofelbamate one fluor atom is attached to the central carbon atom to prevent the formation of the reactive toxic metabolite of felbamamate, ATPAL (Bialer, 2006) (Fig. 5). In carisbamate a chloride atom is attached to the aromatic ring, and an amide containing side chain has been included, resulting in a chiral centrer (Fig. 5).

### Modifications of lamotrigine

#### Parent drug: Lamotrigine

##### Proposed mechanisms of action

The principal mechanism of action of lamotrigine appears to involve inhibition of voltage-activated sodium channels, resulting in increased inhibition of action potential firing activity by a use- dependent mechanism (Xie et al. 1995; Kuo, 1998). Lamotrigine also inhibits high-voltage-activated calcium channels that are located presynaptically, including the N- and P-type, and consequently, inhibits neurotransmitter release, such as glutamate (Xie and Hagan, 1998). Another novel mechanism of action is that lamotrigine selectively decreases action potential firing by an increase in the dendritic hyperpolarization-activated cation current (I_h_), since the dendrites have different electrical properties from the soma in pyramidal cells (Poolos et al. 2002). This target would be of importance in epileptogenesis (Poolos et al. 2002).

##### Clinical use

Lamotrigine is extensively used in epilepsy, neuropathic pain, and bipolar disorder, based on its inhibitory effect on excitatory neurotransmission (Rogawski and Löscher, 2004a, b; Spina and Perugi, 2004; Eisenberg et al. 2005; Nierneberg et al. 2006). In addition, lamotrigine may be beneficial in the treatment of other disorders, such as migraine or schizophrenia (Rogawski and Löscher, 2004a; Lampl et al. 2005; Muzina et al. 2005; Premkumar and Pick, 2006; Johannessen Landmark, 2007a; Anmann et al. 2007).

#### JZP-4

JZP-4, 3-(2,5-trichloro-phenyl)-pyrazine-2,6-diamine, is a derivative of lamotrigine. It is a novel potent sodium and calcium channel inhibitor, which displays broad-spectrum anticonvulsant activity (Bialer et al. 2007). The substance has demonstrated a favorable profile in toxicology and pharmacokinetic studies so far (Bialer et al. 2007).

### Chemical characteristics and modifications of lamotrigine and its derivative

**Figure 6:**
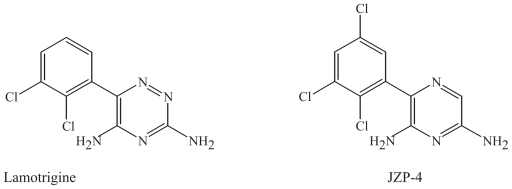
Lamotrigine and its derivative JZP-4.

Lamotrigine is a 1,2,4-triazine, with two chloride atoms attached (Fig. 6). In JZP-4, one nitrogen atom has been removed from the cyclic structure, and another chloride is attached to the aromatic ring (Fig. 6). The two drugs are different in structure since cyclic structures with two nitrogen atoms are commonly occurring in biological molecules, while three nitrogens are not common. Both molecules are neutral in physiological pH. The modifications in JZP-4 may affect the metabolic route of elimination for lamotrigine.

### Comments

In this section, chemical modifications of AEDs, developed to reduce toxic effects, the risks of specific adverse effects and interaction potential have been described. The new derivatives have improved tolerability and pharmacokinetic properties. The main AEDs used today in epilepsy and other disorders as bipolar disorder and neuropathic pain are valproate, carbamazepine and lamotrigine. These drugs have several new effective and safe derivatives that hopefully will be clinically useful within the coming years.

## Improved Efficacy of New AEDs

**Figure 7:**
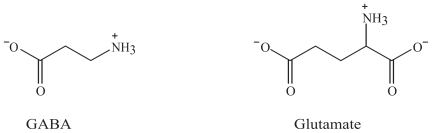
The main inhibitory and excitatory amino acid neurotransmitters in the brain, GABA and glutamate, respectively.

One way to improve efficacy of AEDs is by designing a better pharmacodynamic profiles with more specific mechanisms of action based on new pathophysiological findings. New AEDs in development will hopefully result in better chemical and pharmacological characteristics than the existing substances. The proposed mechanisms of action of these drugs are illustrated in the synapses by the main inhibitory neurotransmitter GABA and the main excitatory neurotransmitter glutamate with their main targets for pharmacological action (Fig. 1). The main pharmacodynamic mechanisms responsible for the clinical efficacy of AEDs include increased GABAergic or decreased glutamatergic neurotransmission, inhibition of voltage-gated ion channels or modifications of intracellular signalling pathways (Rogawski and Löscher, 2004b; Johannessen Landmark, 2007b). A common result of pharmacological intervention with these drugs is a decrease in neuronal excitability. The main modifications of AEDs and their main achievements are listed in Table 1. Most AEDs bind to target macromolecules where the neurotransmitters GABA or glutamate bind, such as receptors and transporters, or to voltage-gated ion channels. Structurally, some of the AEDs may have structural similarities with these amino acid neurotransmitters (Fig. 7), such as gabapentin. Most AEDs have several target molecules in common with the endogenous neurotransmitters (receptors, enzymes, reuptake proteins). In addition, pregabalin and gabapentin are structurally related to GABA.

### Modifications of levetiracetam

#### Parent drug: Levetiracetam

##### Proposed mechanisms of action

Levetiracetam is a unique broad-spectrum AED that did not show any effect in the commonly used epilepsy models, but it was effective in a genetic absence epilepsy model (GAERS) (Bialer et al. 2004, Stratton et al. 2003). A novel binding site for levetiracetam has been identified as the synaptic vesicle protein 2A (SV2A) in the presynaptic neuron (Lynch et al. 2004). It is still unclear how levetiracetam and SV2A interact, but it has been proposed that it is essential for exocytosis of neurotransmitter from the presynaptic neuron into the synaptic cleft and may prevent exocytosis of glutamate, since there is a correlation between binding affinity and potency in suppressing tonic seizures in audiogenic sensitive mice (Lynch et al. 2004). In addition, for other AEDs like gabapentin and pregabalin that target voltage-activated sodium and calcium channels (including α2δ), inhibition of glutamate release is likely to be a critical downstream action for seizure protection (Rogawski and Löscher, 2004a; Rogawski, 2006). Levetiracetam may also influence GABAergic activity by increasing chloride currents and consequently enhancement of inhibitory GABAergic neurotransmission, and glycin could also be affected (Poulain et al. 2002; Rigo et al. 2002). Inhibition of glycin release could indirectly reduce the activity of the NMDA receptor of glutamate, since this receptor is dependant on the binding of both glutamate and glycin to open the ion channel. Recently, enhancement of release of inhibitory neurotransmitters has also been proposed (Stefan and Feuerstein, 2007). It seems more likely, however, that exocytosis is inhibited rather than enhanced when levetiracetam possibly interacts with the docking process of exocytosis of neurotransmitter from the presynaptic neuron.

##### Clinical use

Levetiracetam is used in epilepsy and neuropathic pain and is under investigation for further clinical use, as in essential tremor (Rogawski and Löscher, 2004a; Handforth and Martin, 2004; Bushara et al. 2005; Johannessen Landmark, 2007a). It is extensively used in several seizure types in many patients with refractory epilepsy (Bialer et al. 2007; Stefan and Feuerstein, 2007; Johannessen Landmark et al. 2007).

#### Brivaracetam and seletracetam

Two promising novel drug derivatives of levetiracetam are in developement, brivaracetam (ucb 34714) and seletracetam (ucb 44212). They optimize the unique mechanism of action and may further improve medical management of epilepsy (Klitgaard, 2005; Von Rosenstiel et al. 2007; Bennett et al. 2007). Brivaracetam could possibly have a broader therapeutic spectrum than its parent drug since it also inhibits voltage gated sodium channels (Rogawski, 2006). These derivatives have been tested in phase I studies (Bialer et al. 2007). Comparable studies of the two drugs are, however, lacking, regarding mechanisms of action and efficacy in preclinical models.

### Chemical characteristics and modifications of levetiracetam and its derivatives

**Figure 8:**
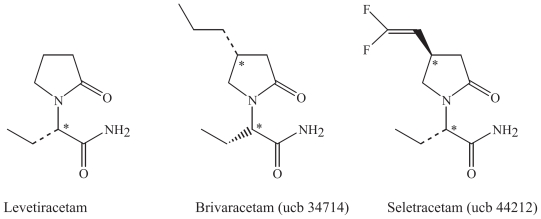
Levetiracetam and its derivatives brivaracetam and seletracetam.

Levetiracetam is a 5-oxopyrolidine with a lactam ring structure with one chiral centre, and the S-enantiomer is present (Fig. 8). Seletracetam and brivaracetam are derivatives of levetiracetam that are substituted at the 4-position in the 2-pyrrolidinone ring (Fig. 8). The chemical difference in the two new compounds is the addition of a propyl group in brivaracetam. In seletracetam, a vinyl group including two fluor atoms is added in the same position as a steric hinder to avoid chemical interactions. All three molecules are neutral in physiological pH. The two derivatives have one additional chiral centre, giving two stereoisomers each. Stereoselectivity in the binding of these molecules seems likely to occur. These modifications result in a potentiated binding to the SV2A protein, up to 10-fold compared to levetiracetam in several models for epilepsy, like cornea kindling and the GAERS absence seizure model (Crowder et al. 1999, Lynch et al. 2004, Bennett et al. 2007, Von Rosenstiel et al. 2007).

### Modifications of gabapentin

#### Parent drug: Gabapentin

##### Proposed mechanisms of action

Gabapentin was synthesized as a GABAergic substance and is structurally related to GABA, but does not interact with GABA receptors or its uptake or degradation processes (Sills, 2006). Gabapentin and pregabalin are ligands of α2δ (1 and 2) voltage-activated calcium channel subunits that are over-expressed in sensory neurons after nerve injury (Marais et al. 2001; Bian et al. 2006). Inhibition of voltage-gated calcium channels in the presynaptic neuron will inhibit glutamate release at excitatory synapses and thereby a decrease in excitatory neurotransmission (Rogawski and Löscher, 2004a).

#### Pregabalin

A structurally related compound to gabapentin is pregabalin, (S)- 3-aminomethyl-5-methylhexanoic acid. Pregabalin has the same binding-affinity as gabapentin to the α2δ(1 and 2) subunits, which strongly implicates that these subunits are important for the pharmacological effect (Marais et al. 2001; Bian et al. 2006). The subunits are major binding proteins for pregabalin in neocortex, hippocampus, amygdala, and spinal cord, as demonstrated in genetically modified mice (Bian et al. 2006). It remains to be determined, however, whether an interaction with high-voltage-activated calcium channels is sufficient to account for the broad-spectrum activity of gabapentin and pregabalin (Sills, 2006).

##### Clinical use of gabapentin and pregabalin

Gabapentin and pregabalin have recognized efficacy in the treatment of both epilepsy and neuropathic pain, and to some extent in migraine, and they are both marketed in many countries (Landy et al. 2004; Sills, 2006). The inhibition of voltage-gated calcium channels is supposed to be involved in pain relief (Marais et al. 2001; Rogawski and Löshcer, 2004a, Sills, 2006; Bian et al. 2006). Gabapentin has also demonstrated efficacy in essential tremor, which is associated with a deficiency in GABAergic function (Kralic et al. 2005; Jankovic and Noebels, 2005; Rodrigues et al. 2005).

#### XP13512

A new derivative is in development as a prodrug of gabapentin, XP13512, [(+/−)-1-([(alpha-isobutanoyloxyethoxy)carbonyl] aminomethyl)-1-cyclohexane acetic acid], avoiding capacity-limited absorption from the intestine following oral administration (Cundy et al. 2004; Bialer, 2006). It is going through phase II in clinical trials (Bialer, 2006). Its potential clinical use will possibly be primarily epilepsy and neuropathic pain.

### Chemical characteristics and modifications of gabapentin and its derivatives

**Figure 9:**
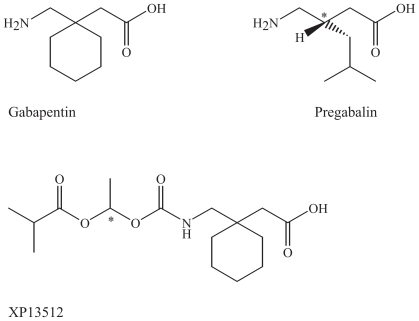
Gabapentin and its derivatives pregabalin and XP13512.

The structures of gabapentin and pregabalin are derived from GABA, but they are one atom longer, with six instead of five carbon or nitrogen atoms in a row. Both drugs are inactive at GABA receptors (Taylor et al. 2006) (Fig. 9). Unlike GABA both drugs have bulky aliphatic chemical substitutions at the 3-position of GABA, which changes their pharmacological properties significantly in comparison to GABA (Taylor et al. 2006). In gabapentin, there is an amino acid-like structure with a carboxylic acid ending and one amino group ending, attached to a cyclohexane (Fig. 9). In pregabalin, there is a central chiral atom in the 4-position, and the amino acid-like structure is kept. The cyclic hexane structure has been replaced with an aliphatic side chain. These aliphatic amines are positively charged at physiological pH. The gabapentin derivative XP13512 has got an addition of a diesther group to the amino ending of the molecule (Fig. 9).

### Compounds related to topiramate

#### Parent drug: Topiramate

##### Proposed mechanisms of action

Topiramate is a pharmacologically rich neuroactive drug that has been demonstrated to possess many molecular activities, such as inhibition of voltage-gated sodium channels, modulation of voltage-gated potassium and calcium channels, modulation of GABA_A_ and glutamate receptors, and carbonic anhydrase inhibition (Bialer et al. 2004; White, 2005). Among its complex actions, topiramate is a selective antagonist at the glutamatergic kainate receptor, a potential and important target regarding minimizing the potential toxicity caused by prolonged glutamatergic activation by NMDA receptors (Kaminski et al. 2004; Muir, 2006). It is rather remarkable that the unusual structure of topiramate may affect these distinct targets including receptors, ion channels and enzymes.

##### Clinical use

Topiramate is used in epilepsy, migraine and tremor (Silberstein et al. 2004; White, 2005; Bialer et al. 2007). Topiramate may have a potential for clinical use in treatment-resistant schizophrenia, even though the outcome was moderate in one clinical study (Tiihonen et al. 2005). Furthermore, the drug has been tested in other neurological and psychiatric conditions, such as anxiety, bipolar disorder, and neuropathic pain (Van Ameringen et al. 2004; Bartolini et al. 2005; McIntyre et al. 2005; Johannessen Landmark, 2007a).

The following compounds are not structurally related to topiramate, but are compounds with a similar mechanism of action compared to some of the actions mediated by topiramate, e.g. antagonists to glutamatergic and GABAergic receptors. Efforts have been focused on improving the selectivity of potential AEDs to these receptors.

#### Talampanel

It has been difficult to develop antagonists to ionotropic glutamate receptors, both due to the high endogenous extracellular glutamate concentration and the possible side effects on cognition and memory. Talampanel is a selective non-competitive antagonist at the AMPA receptor (Rogawski, 2006; Solyom et al. 2002). Talampanel has been suggested to be active in neuroprotection as well as in epilepsy (Bialer, 2006). Two putative noncompetitive antagonist sites are known. These binding sites are linked to the cation channel of the AMPA receptor (Bialer et al. 2007). Clinical trials of talampanel are ongoing in phase II (Bialer et al. 2007).

#### NS1209

NS1209 is a selective, competitive AMPA receptor antagonist (Rogawski, 2006; Solyom et al. 2002). AMPA receptors are key mediators for seizure spread. Calcium permeability of AMPA receptors lacking the GluR2 subunit in the amygdala and hippocampus has been studied, and they could play a role in synaptic plasticity, epileptogenesis and excitotoxicity (Gryder et al. 2005). These receptors are promising targets for further investigations. There is a recognized glutamate binding site on the AMPA receptor subunit, where competitive antagonists like NS1209 bind.

### Chemical characteristics and modifications of topiramate and related compounds

**Figure 10:**
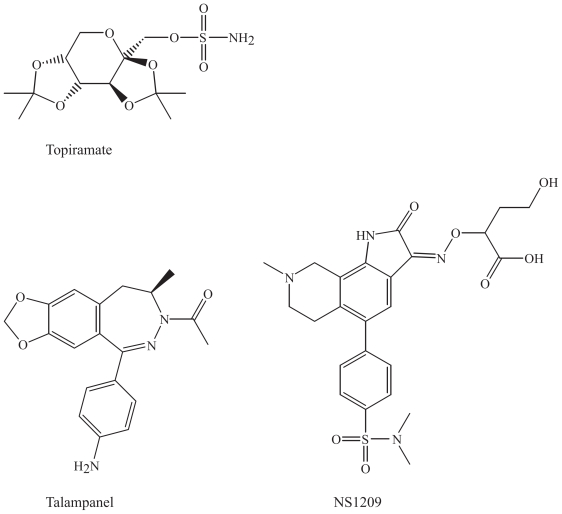
Topiramate and compounds with similar mechanism of action at the AMPA receptor.

The following structures are not very similar chemically but bind to a common target protein, the AMPA receptor. Topiramate is a tetrahydropyrane with one sulphonamide attached. Two acetal groups with two methyl groups each are attached to the molecule to protect the substituents (Fig. 10). Talampanel is a diazepine derivative, the 3-acetylated, 3-4-dihydro analogue of GYKI 52466, which is the prototype of the 2,3-benzodiazepine class of AMPA receptor antagonists (Donevan and Rogawski, 1993). NS1209 is an indole derivative included in a big heterocyclic structure with several attached groups, including one sulphonamide group (Fig. 10). They are large heterogenous molecules and differ in functional groups, which indicate that the whole molecule is not involved in the binding for pharmacological effect.

### Benzodiazepines, neurosteroids and their derivatives: GABA**A** agonists

#### Parent drugs: Benzodiazepines

##### Proposed mechanisms of action

The benzodiazepines bind to the benzodiazepine receptor within the benzodiazepine-GABA_A_ receptor complex. Agonists at the GABA_A_ receptor enhance the inhibitory GABAergic neurotransmission throughout the CNS. The various GABA_A_ receptor subtypes are different in their specific regional and cellular localization, they consequently serve distinct neuronal circuits and functions. A deficit in the α_3_-subunit in GABA_A_ receptors was linked to dopaminergic hyperfunction, which is considered to be a contributing factor to the development of schizophrenia, and agonists at these receptors would be of importance (Yee et al. 2005).

##### Clinical use

Benzodiazepines are used worldwide as anxiolytics, sedatives and anticonvulsant drugs. The potential for development of tolerance, dependence and abuse, is, however, a limitation in their clinical use (Löscher and Schmidt, 2006). The new derivatives may have a clinical potential in anxiety and epilepsy.

#### ELB139

Various selective partial benzodiazepine receptor agonists are being developed, including ELB139, [1-(4-chlorophenyl)-4-piperidin-1-yl-1,5-dihydro-imidazol-2-on], a positive allosteric modulator of α3-containing GABA_A_ receptors (Langen et al. 2005). In a recent study, ELB139 showed functional subtype specificity to several GABA_A_ receptor subtypes, and partial agonism at various subtypes, differently from diazepam (Rabe et al. 2007). Its main clinical use seems to be anxiety, although it may have a potential in epilepsy (Rogawski, 2006).

#### Ganaxolone

**Figure 11:**
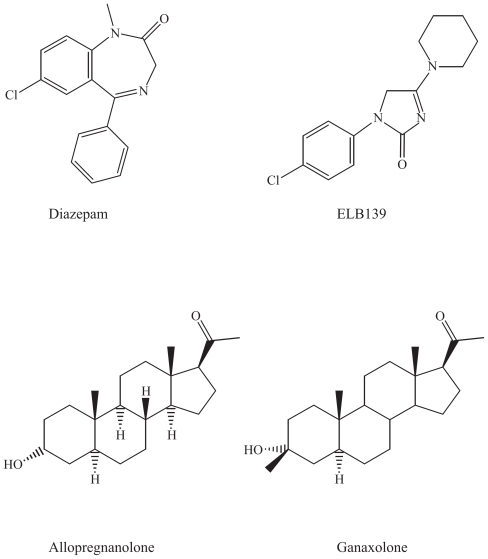
Agonists at the GABA_A_ receptor, benzodiazepines and progesterone derivatives.

Ganaxolone (3-α-hydroxy-3-β-methyl-5-α-pregnan-20-on) is the 3-β-methylated synthetic analogue of allopregnanolone (Fig. 11). It is a neuroactive progesterone-analogue that acts as a positive modulator of GABA_A_ receptors, since there is a specific binding site for these pregnanolones (Foster and Kemp, 2006). It is a member of a novel class of neuroactive steroids called epalones, and it allosterically modulates the GABA_A_ receptor complex via a unique recognition site (Bialer et al. 1999). Ganaxolone has demonstrated efficacy in preliminary clinical trials, in infantile spasms in children and as monotherapy in adults (Kerrigan et al. 2000; Laxer et al. 2000). It is currently undergoing further development in infantile spasms, in women with catamenial epilepsy, and in adults with refractory partial-onset seizures (Nohria and Giller, 2007). The potential for clinical use of ganaxolone seems to be rather limited based on the absence of recent publications of further investigations regarding this drug, possibly due to pharmacokinetic problems and limited absorption, and reformulation of the drug is ongoing (Bialer et al. 1999; Nohria and Giller, 2007).

### Chemical characteristics and modifications of benzodiazepines, neurosteroids and their derivatives

#### GABA_A_-agonists

Chemically, ELB-139 is related to the benzodiazepines, as both drugs consist of heterocyclic ring structures that possibly bind similarily to the GABA_A_ receptor, although this new derivative is subtype selective.

#### Neurosteroids

Ganaxolone has a steroid skeleton very similar to progesterone, with a difference in the hydroxyl group instead of a keton and a double bond in the A-ring of the cholesterol skeleton (Fig. 11). It lacks, however, hormonal activity (Bialer et al. 1999).

### Comments

In this section important target molecules for AEDs have been discussed in relation to the different classes of AEDs. The molecules that have been focused upon include voltage gated sodium and calcium channels, proteins associated with exocytosis of neurotransmitters (SV2A), and receptor molecules for GABA (GABA_A_) and glutamate (AMPA receptors). Further investigations with potential AEDs should be accompanied by pharmacogenetic studies to elucidate modifications in the target molecules of AEDs, which also may be altered in pathophysiological processes (as in anxiety with altered subunit expression of the GABA_A_ receptors and neuropathic pain where an over-expression of the α2δ subunits of the voltage-gated calcium channel is present).

## Conclusions

Several AEDs of a new generation have been developed from the existing drugs. Develoment of new derivatives is important to achieve new drugs with improved pharmacokinetic and—dynamic properties, resulting in better tolerability and efficacy. The AEDs consist of a group of heterogenous chemical structures, unrelated to each other, but affecting the same target proteins. Important target molecules, which have been focused upon include voltage gated sodium and calcium channels, proteins associated with exocytosis of neurotransmitters, and receptor molecules for GABA and glutamate. New drugs will hopefully affect pathophysiological processes or altered target proteins more selectively than older drugs. Further effort should be put into more detailed structure-activity relationship studies including investigations regarding stereoselectivity. There will be a resistant need for improvement of efficacy and tolerability of the existing drugs, as well as newly discovered substances with novel mechanisms of action may become of importance in future drug therapy in epilepsy in addition to other CNS disorders.

## Figures and Tables

**Table 1:** A new generation of existing AEDs, their parent compound and improvements.

New compound	Parent AED	Improvement	Characteristics
Brivaracetam Seletracetam	Levetiracetam	Efficacy	More potent binding to SV2A
Oxcarbazepine Eslicarbazepine	Carbamazepine	Tolerability	Improved pharmacokinetic
Fluorofelbamate	Felbamate	Tolerability	Non-toxic metabolite
JZP-4	Lamotrigine	Efficacy	Improved pharmacokinetic properties and efficacy
Pregabalin	Gabapentin	Efficacy	Equally potency to gabapentin
Valrocemide, Valnoctamide, Propylisopropyl acetamide (PID), N-methyl-2,2,3,3-tetramethylcyclopropylamide (MTMCD), NPS-1776, Tetramethyl-cyclopropancarbonylurea (TMCU)	Valproate	Tolerability	Less toxic metabolites, less teratogenic potential
